# Detecting Avascular Necrosis of the Lunate from Radiographs Using a Deep-Learning Model

**DOI:** 10.1007/s10278-023-00964-0

**Published:** 2024-01-16

**Authors:** Krista Wernér, Turkka Anttila, Sina Hulkkonen, Timo Viljakka, Ville Haapamäki, Jorma Ryhänen

**Affiliations:** 1 Musculoskeletal and Plastic Surgery, Department of Hand Surgery, University of Helsinki and Helsinki University Hospital, Haartmaninkatu 4, 00290 Helsinki, Finland; 2 Tampere University Hospital, Tampere, Finland; 3 Department of Radiology, HUS Diagnostic Center, HUS Medical Imaging Center, Helsinki, Finland

**Keywords:** Artificial intelligence, Osteonecrosis, Deep learning, Kienbock disease, Diagnosis, Bone avascular necrosis

## Abstract

Deep-learning (DL) algorithms have the potential to change medical image classification and diagnostics in the coming decade. Delayed diagnosis and treatment of avascular necrosis (AVN) of the lunate may have a detrimental effect on patient hand function. The aim of this study was to use a segmentation-based DL model to diagnose AVN of the lunate from wrist postero-anterior radiographs. A total of 319 radiographs of the diseased lunate and 1228 control radiographs were gathered from Helsinki University Central Hospital database. Of these, 10% were separated to form a test set for model validation. MRI confirmed the absence of disease. In cases of AVN of the lunate, a hand surgeon at Helsinki University Hospital validated the accurate diagnosis using either MRI or radiography. For detection of AVN, the model had a sensitivity of 93.33% (95% confidence interval (CI) 77.93–99.18%), specificity of 93.28% (95% CI 87.18–97.05%), and accuracy of 93.28% (95% CI 87.99–96.73%). The area under the receiver operating characteristic curve was 0.94 (95% CI 0.88–0.99). Compared to three clinical experts, the DL model had better AUC than one clinical expert and only one expert had higher accuracy than the DL model. The results were otherwise similar between the model and clinical experts. Our DL model performed well and may be a future beneficial tool for screening of AVN of the lunate.

## Introduction

Avascular necrosis (AVN) of the lunate, also known as Kienböck disease or lunate malacia, is a rare condition that mostly affects men aged between 20 and 40 years [1]. As the exact etiology, pathomechanisms, and natural progression of the disease remain unclear, the most appropriate treatment remains under debate [2–4]. Symptoms typically vary between patients. Detection from plain radiographs is not possible in the early stage [5], which can lead to delayed diagnosis. Consequently, fewer treatment options are available, and surgical procedures at this point may be limited to procedures, which restrict range of motion and hand performance [6, 7]. This can be particularly challenging for manual workers, who are also more susceptible to the disease [8].

According to the Lichtman classification, AVN of the lunate is categorized from radiographs into stages I–IV [9]. Stage I can only be diagnosed with MRI, while later stages are also visible in radiographs. It would be beneficial to develop a diagnostic tool to aid in earlier diagnosis and hopefully create a better understanding of the disease’s natural progression from a potentially asymptomatic condition to possible lunate collapse and symptomatic arthrosis. An accurate deep-learning (DL) model could be especially helpful as a screening tool to identify suspicious cases already in primary health care.

DL algorithms are rapidly becoming key instruments in medical imaging. In particular, significant advances of convolutional neural networks (CNN) in image classification and object detection have enabled new tools in medical diagnostics [10]. In many instances, such tools have already achieved a human-level accuracy in detecting abnormalities from both radiographs and photographs [11–13].

This study aimed to determine if our DL algorithm can recognize AVN of the lunate from wrist radiographs and compare the results to those obtained from clinical experts.

### Patients and Methods

**Fig. 1:**
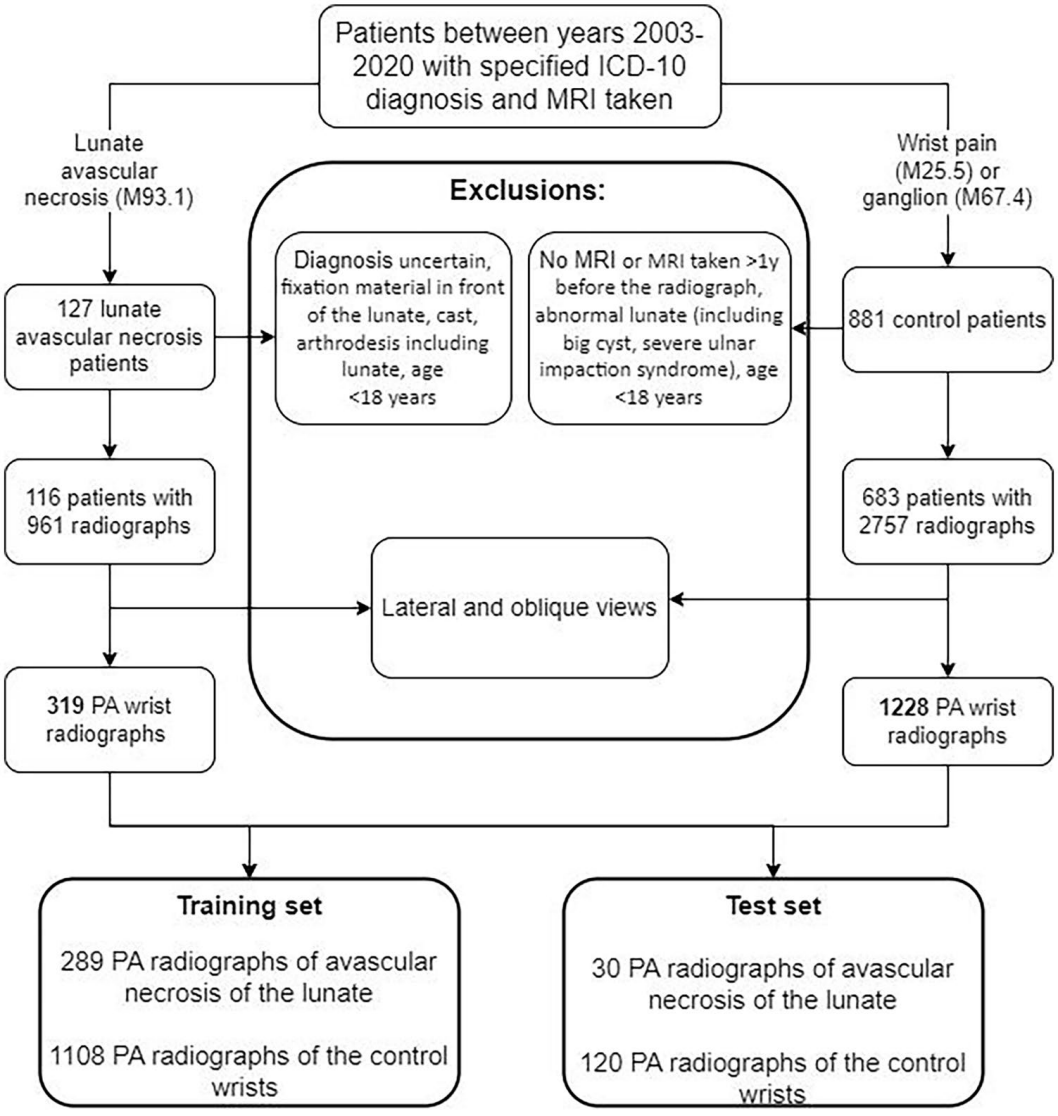
Data collection flowchart. PA, postero-anterior; MRI, magnetic resonance imaging; M93.1, M25.5, M67.4, diagnosis codes from International Classification of Diseases, ICD-10

Patients with AVN of the lunate and control patients were identified from the Helsinki University Central Hospital database between 2003 and 2020 with International Classification of Diseases (10th revision, ICD-10) codes M93.1, M25.5, and M67.4. Figure 1 shows the data collection procedure and exclusion criteria. For controls, patients with diagnosis of joint pain (M25.5) or ganglion (M67.4) and available magnetic resonance imaging (MRI) of the wrist were identified. Altogether, 127 AVN patients and 881 controls were evaluated. All MRIs were assessed by radiologists. A hand surgery resident (K.W) and a hand surgeon (T.A) also examined all medical reports, MRIs, and radiologist reports for AVN to confirm diagnosis and for controls to confirm normal vasculature of the lunate.

Patients with mild ulnar impaction syndrome and postoperative AVN patient radiographs with no hardware on top of the lunate were included in the study. For controls, radiographs taken before or within a year after clean MRI were included to minimize the risk of the patient potentially having abnormalities in the lunate vasculature and thus affecting DL model training. For the AVN cohort, all radiographs were assessed and categorized into stages I–IV according to the Lichtman classification [9]. Unclear cases (diagnosis or staging) were assessed in conjunction with three hand surgeons and one musculoskeletal radiologist. Radiographs from symptomatic patients were considered as stage I when taken at most 1 year before confirmed AVN of the lunate diagnosis in the MRI. A total of 116 AVN cohort patients with 319 postero-anterior wrist radiographs and 683 control cohort patients with 1228 control postero-anterior wrist radiographs were included in the study.

A data analyst subsequently pseudonymized the radiographs and converted the image format from DICOM to PNG. A test set (10% of radiographs) was separated to validate the developed DL model. It was confirmed by the file name that all the radiographs from the same patient were either in the training or the test set. The test set included radiographs from 13 AVN patients and from 78 control patients. Table 1 shows the number and distribution of different stages according to Lichtman classification in both the training and the test set groups. Altogether, 82 (28%) AVN cohort and 290 (26%) control radiographs lacked pixel size, and these were extrapolated from the average number of the known ones.

**Table 1:** Number of patients and radiographs per stage. Staging according to Lichtman [9]

	Training set		Test set	
	Patients	Radiographs	Patients	Radiographs
Stage I	19	42	1	2
Stage II	39	84	5	12
Stage IIIA	42	72	4	8
Stage IIIB	31	64	5	7
Stage IV	12	25	1	1
Total	103	287	13	30

After pseudonymization, the radiographs were exported to a cloud-based artificial intelligence development environment Aiforia Create version 5.5 (Aiforia Technologies Plc, Helsinki, Finland) where the neural network was developed. The model was first trained to recognize the carpal bones. After the carpal bones were detected with high sensitivity, a “child” layer with another independent neural network was trained to detect and segment the lunate as healthy or diseased at the pixel level. Based on the previous classification with MRI, the lunates were annotated to the radiographs as diseased or healthy. Aiforia AI engines use convoluted neural networks for the AI model development.

The following parameter settings were set to train the algorithm: semantic segmentation (region type detection) with field of view 150 µm and complexity level “extra complex” was used for the carpal bone layer and 50 µm field of view with complexity level “extra complex” was used for the lunate layer. Both layers had default image augmentation settings. The default augmentation settings were as follows: scale (−1 to 1.01), aspect ratio (1), maximum shear (1), luminance (−1 to 1.01), contrast (−1 to 1.01), maximum white balance (1), noise (0), JPG compression quality (40 to 60) and rotation angle (−180 to 180), and blur maximum pixels (1) with flipping option set to enabled. For the additional training hyperparameters, initial learning rate was set to 1 with mini-batch size set to 20 along with initial learning rate set to 1. The performance of the resulting model was evaluated visually, from the verification error rates and from the small validation set of 3 images. Finally, the model with 1000 iterations was used for the validation in the test set, and the ROC curve was created with multiple gain values. F1 score for the training set was 90.81%.

The test set radiographs were also examined in the cloud-based environment by clinical experts (two hand surgeons with > 20 years of experience and one musculoskeletal radiologist), and lunate was judged as healthy or diseased. The images were set in random order. The experts performed the assessment separately and were unaware of the correct diagnosis, results of the DL model, or the assessments of the other experts. Interrater agreement was calculated with mean Cohen’s *κ* between different evaluators. The level of agreement was interpreted as suggested by Mary L McHugh [14].

Confidence intervals (CI) for sensitivity, specificity, and accuracy were calculated by the Clopper-Pearson exact method. Likelihood ratios were calculated by the Log method [15]. Predictive values were calculated by the standard logit method by Mercaldo and colleagues [16]. Receiver operating characteristic (ROC) curve analysis was used to assess the overall accuracy and discrimination ability of the DL model and clinical experts. Ninety-five percent CIs for areas under the curves and their statistical comparison were calculated with DeLong’s test. *P*-values < 0.05 were considered statistically significant. ROC curve for the DL model was calculated by varying the model’s gain parameter.

## Results

**Fig. 2:**
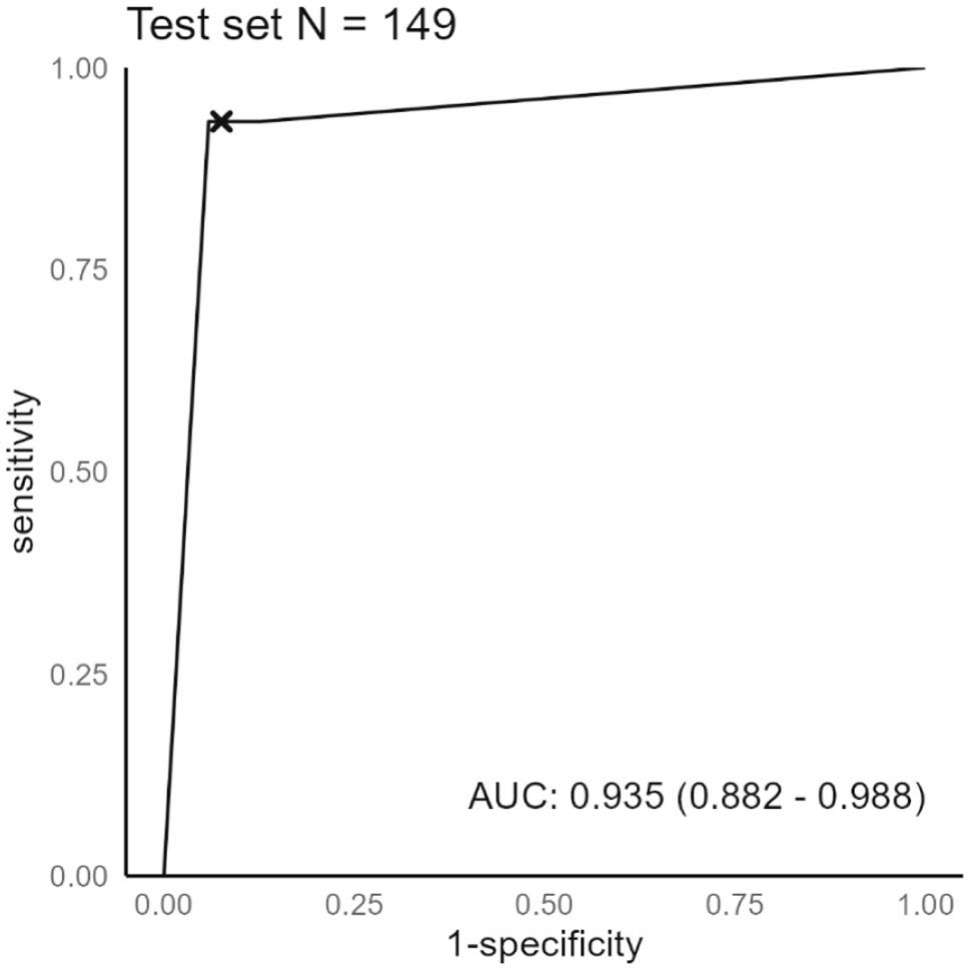
ROC curve indicating DL model performance in the test set

**Fig. 3:**
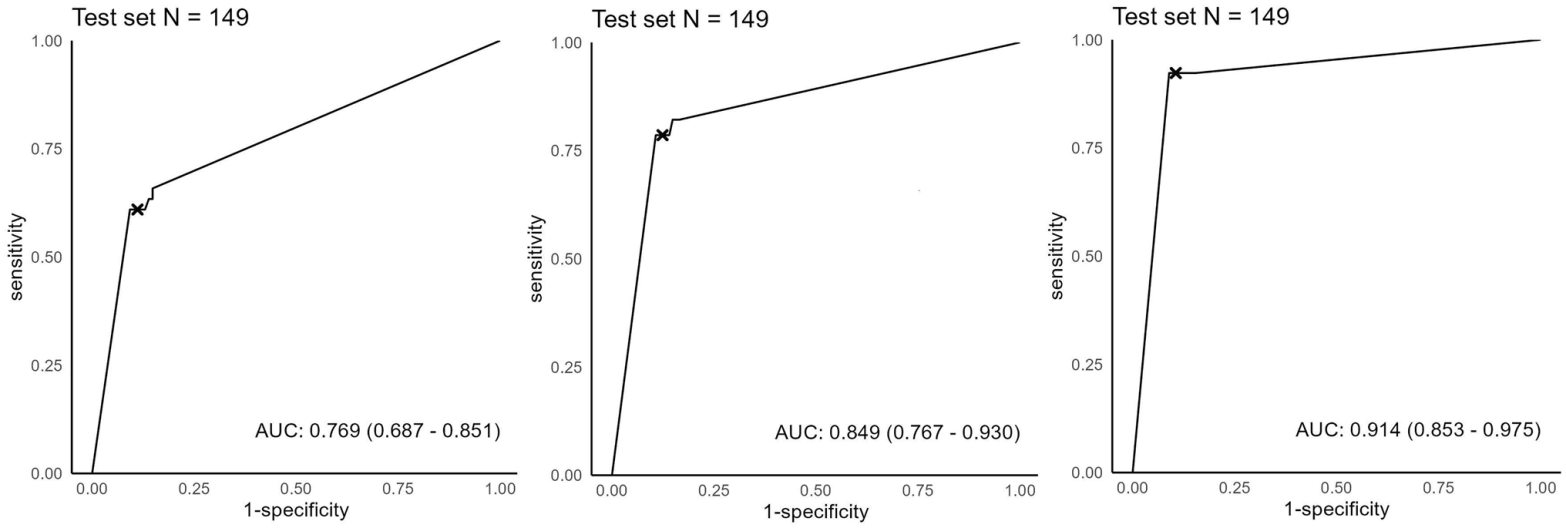
ROC curves of clinical experts indicating discrimination performance in the test set. From the left: surgeon 1, surgeon 2, radiologist

Our DL model recognized AVN of the lunate in 28 out of 30 radiographs in the test set with a sensitivity of 93.33%, specificity of 93.28%, and accuracy of 93.28% for the model. The area under the ROC curve was 0.94 (Fig. 2)*.* ROC curves of clinical experts are presented in Fig. 3. A statistically significant difference of the AUC was found between the DL model and surgeon 1 and between radiologist and surgeon 1 (Table 2). F1 score for the test set was 84.85%.

**Table 2:** AUC comparison with DeLong’s test. Statistically significant P-values are highlighted in bold

Comparison	*P*-value
DL model vs surgeon 1	**0.00**
DL model vs surgeon 2	0.08
DL model vs radiologist	0.62
Surgeon 1 vs surgeon 2	0.18
Surgeon 1 vs radiologist	**0.01**
Surgeon 2 vs radiologist	0.21

**Fig. 4:**
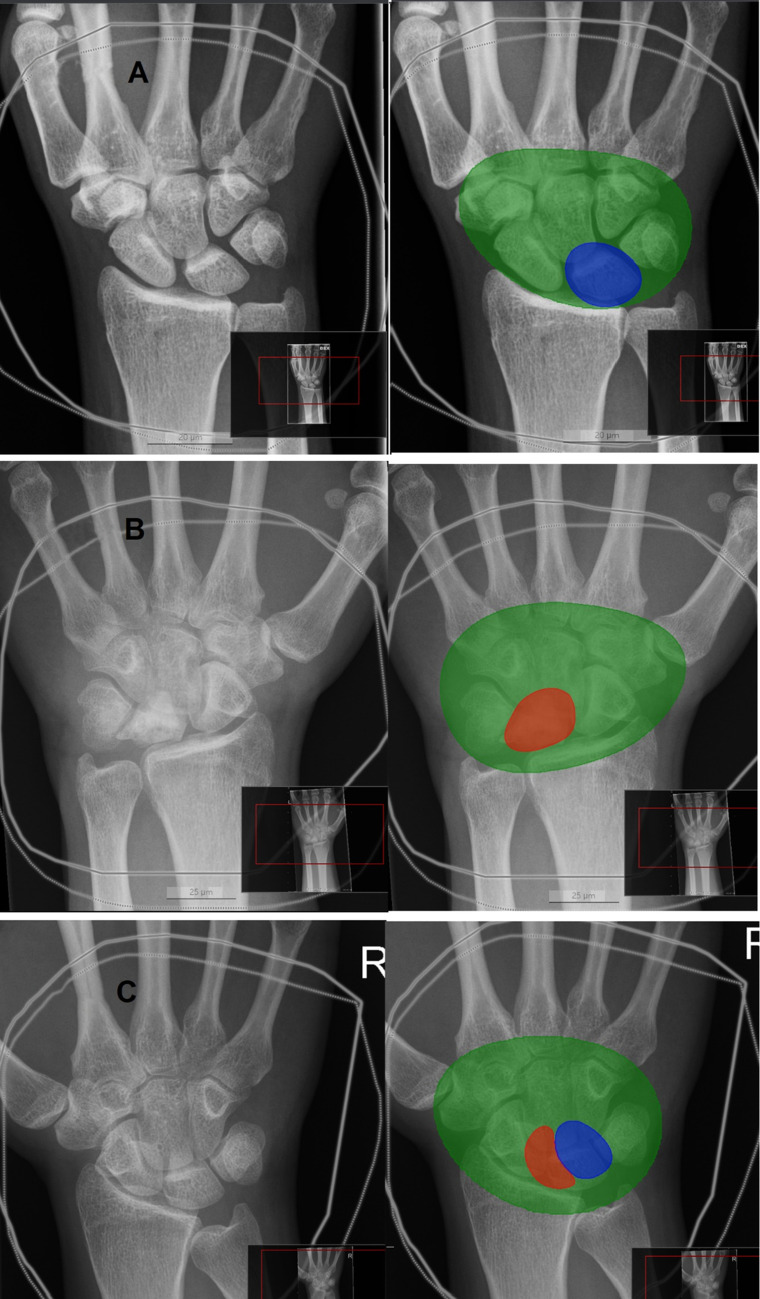
Examples of DL model analyses. Carpal bones are indicated in green, healthy lunate as blue, and avascular necrosis as red. The DL model made the correct analyses in images **A** and **B**. Image **C** represents a control case. As > 30% of the lunate is red, the analysis was interpreted as false positive

**Fig. 5:**
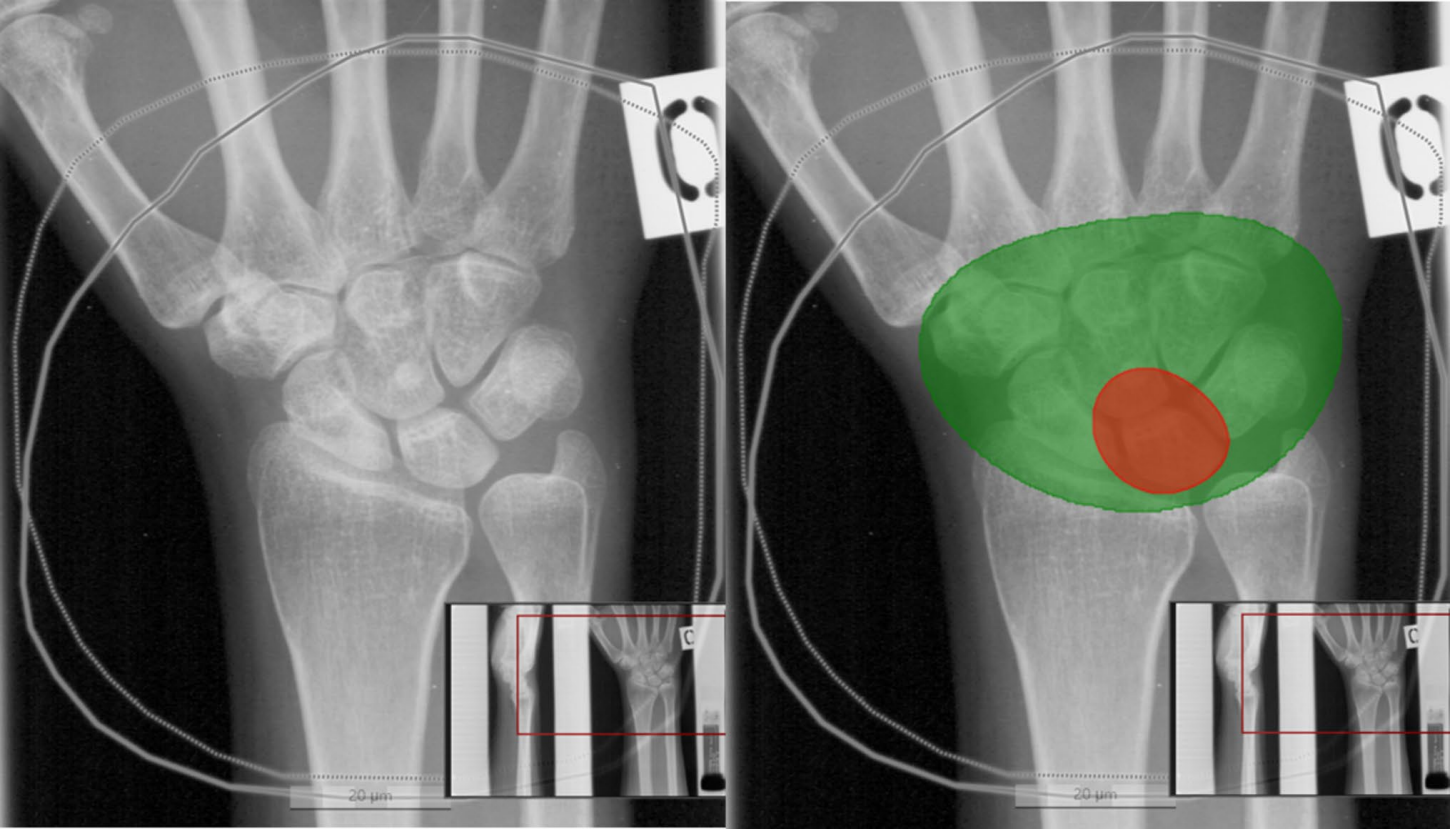
Figure indicates how the DL model recognizes avascular necrosis of lunate stage I from the radiograph that cannot be recognized by human assessment

The true pixel size was missing and was thus extrapolated from the average size in one out of two radiographs that the DL model missed. Both misdiagnosed radiographs were taken from the same patient, but the model correctly recognized the one remaining radiograph from the same patient. Both were stage IIIB and correctly recognized by clinical experts. The DL model identified both stage I radiographs in the test set. The DL model recognized the carpal bones in all except one radiograph. This radiograph was cropped such that only part of the carpal bones was visible and did not for this reason entirely represent a typical wrist radiograph. This was excluded from the performance calculations, as the DL model did not provide any assessment of the lunate. In two radiographs, the carpal bone assessment was too wide, but the DL model recognized the lunate correctly. These radiographs were quite dark. There was a partial false positive/negative segmentation either in the lunate or somewhere else in the radiograph in 10 control and 1 cohort cases. An example is shown in image C in Fig. 4. The assessment was considered as AVN if > 30% of the lunate was detected by the DL model as diseased bone. Figure 5 shows an example of the DL model’s recognition of stage I disease. The extrapolated pixel size in control radiographs coincided with lunate drawings out of shape. The number of false-positive radiographs was eight; four of these had extrapolated pixel size. Table 3 shows the test set confusion matrix, and Table 4 shows the overall results with 95% CIs.

**Table 3:** Test set confusion matrix

	Avascular necrosis of the lunate	Healthy control
Test positive	28	8
Test negative	2	111
Total	30	119

**Table 4:** Results for the test set

Statistic	Value (95% CI)
AUC	0.94 (0.88–0.99)
Sensitivity	93.33% (77.93–99.18%)
Specificity	93.28% (87.18–97.05%)
Accuracy*	93.28% (87.99–96.73%)
LR +	13.88 (7.06–27.30)
LR−	0.07 (0.02–0.27)
Positive predictive value*	3.62% (1.88–6.88%)
Negative predictive value*	99.98% (99.93–99.99%)

*Exact prevalence unknown, estimate 0.27% used in the calculations is based on van Leeuwen W et al. et al. [27]

Interrater agreement was moderate (*κ* = 0.715; 95% CI 0.581–0.849; *P* < 0.001). A comparison of the performance of the DL model and clinical experts with 95% CIs is shown in Table 5. The only statistically significant difference was between the musculoskeletal radiologist and the DL model in accuracy. The radiologist also acquired statistically significantly higher accuracy than both surgeons and better specificity than one surgeon.

**Table 5:** Results of the DL model compared with clinical experts. *DL* deep learning

	Sensitivity (95% CI)	Specificity (95% CI)	Accuracy (95% CI)*
Surgeon 1	90.00% (73.47–97.89%)	88.33% (81.20–93.47%)	88.34% (82.09–93.00%)
Surgeon 2	80.00% (61.43–92.29%)	96.67% (91.69–99.08%)	96.62% (92.33–98.88%)
Radiologist	86.67% (69.28–96.24%)	100.00% (96.97–100.00%)	99.96%
DL model	93.33% (77.93–99.18%)	93.28% (87.18–97.05%)	93.28% (87.99–96.73%)

*Estimated prevalence 0.27% [27] was used in the calculations

## Discussion

Our DL model detected AVN of the lunate with promising accuracy and may be a useful diagnostic tool in the future. Based on the observed sensitivity and specificity, the current DL model missed only 6.7% of the presented radiographs with AVN and misinterpreted healthy ones as diseased in 6.7% cases. Furthermore, the model had a higher AUC than one clinical expert.

The musculoskeletal radiologist was better than experienced hand surgeons and the DL model in accuracy but not in sensitivity and had a higher AUC and specificity than one of the hand surgeons. This is an interesting observation and underlines the fact that only radiologists have received education in image interpretation. The biggest benefit of the DL model would probably be in aiding diagnosis in primary health care, where reports of radiographs often come late and from radiologists not specialized in musculoskeletal imaging.

To our knowledge, there are no previous studies that have investigated use of DL algorithms in identifying AVN of the lunate. However, the use of DL algorithms has been reported in the detection and classification of osteonecrosis of the femoral head from both radiographs and MRIs. These studies show that it is possible to achieve results comparable to experienced clinicians [17–21]. Sensitivity of the algorithms was lower in the external test sets [17, 19], which underlines the need for external validation of the algorithms. Li et al. [20] achieved statistically significantly higher sensitivity for their model when they combined AP and lateral view radiographs instead of using only one view. This would be interesting to test further in our data.

Anttila et al. [22] investigated the use of a segmentation-based DL model in detection of distal radius fractures and obtained an AUC of 0.97 (95% CI 0.95–0.98) for the model. Ashkani-Esfahani et al. [23] used transfer learning in adopting pretrained CNNs to assess presence of ankle fracture in the radiograph. The use of pretrained algorithms may improve the performance of the DL models. The weakness of these studies when compared with ours is the less rigorous ground truth labeling of presence or absence of disease. We have MRIs of all control patients to exclude the possibility of AVN. CT would be needed for every patient to exclude fractures not visible in radiographs. Lindsey et al. [24] demonstrated that assistance from DL algorithms significantly improved fracture detection of clinicians. They had multiple approaches to reduce overfitting, including bootstrapping stage, early stopping, and data augmentation. Nonetheless, they preprocessed the radiographs and rescaled them to a fixed resolution, which may change the performance of the model in an actual clinical setting.

Surprisingly, our model recognized Lichtman stage I from radiographs, which is not by definition visible to the human eye. Previously, David W. G. Langerhuizen et al. [25] presented their DL algorithm that recognized five out of six occult scaphoid fractures that were missed by human observers. Detection of changes from radiographs that are not visible to the human eye is very intriguing. If this performance is achieved in clinical practice, the DL model could be extremely useful in future diagnostics of AVN of the lunate. There are more treatment options the earlier the disease is diagnosed. Especially for general practitioners and non-specialized radiologists, it would be beneficial to have this kind of tool to raise suspicion of disease sooner and expedite referral to a hand surgeon.

There is unfortunately minimal published data on the epidemiology of AVN of the lunate [26–29] and no literature about the prevalence in Finland. Accordingly, calculations of positive predictive value (PPV) and negative predictive value (NPV) are rough estimates. Nevertheless, AVN of the lunate is known to be rare, and thus PPV would probably be somewhat low and result in a greater number of false positives. This would, in turn, result in higher costs if refuting the positive findings with MRI and may potentially cause unnecessary concern for patients. For a rare condition such as AVN of the lunate, where the pre-test likelihood is very low and the clinical resources available for additional diagnostic imaging are limited, medical authorities would need to decide on the optimal threshold where the DL model can be implemented in clinical practice with optimal benefit for society and the patient.

Our study had some limitations, in particular the small sample size that is due to the rarity of the disease. However, the study population consisted of all patients with AVN of the lunate treated in Helsinki University Hospital between 2003 and 2020, with radiographs available for research purposes. Accordingly, there was a low risk of sampling bias. Another limitation is the lack of external validation for which data were unavailable. External validation of the model must be performed before any possibility of clinical implementation. This study should be considered as proof of concept. Unfortunately, there is no state-of-the-art method to diagnose AVN of the lunate except for clinical assessment from radiographs or MRI. Therefore, comparison of the model has only been made against a clinician’s assessment. We did not include patients with severe ulnar impaction syndrome and large lunate cysts as controls, which might have confused the model. These conditions may increase the number of false positives in real-life practice. We included radiographs taken after different operations or with hardware in place except when directly on top of the lunate. In eight cases (21 radiographs), the stage remained as I after the operation and in one case it improved from stage II to I. In one case (6 radiographs), the operation was done as a correction osteotomy after distal radius fracture and not because of AVN of the lunate. MRI was not repeated after the operations, and hence there is no way of knowing whether the AVN could have healed. This may have resulted in a few false positives in stage I images. The absence of some pixel sizes in the radiograph metadata seemed to affect the precision of the DL model in some cases. After initial results, most of the missed AVN radiographs seemed to have the extrapolated pixel size. A few exact pixel sizes were found afterwards; the DL model recognized AVN better when these were corrected. Therefore, the overall results may have been affected if all pixel sizes were available.

The challenge of overfitting emerges when a neural network boasts an excessive number of layers or when the training dataset is inadequately small. This results in an AI model that becomes excessively precise within the confines of the training data, rendering it unsuitable for the generalization of results in subsequent analyses. In our case, the disease in question is a rarity, making it impractical to amass a substantial volume of data. Nevertheless, we sourced data from several hospitals within the Helsinki University Hospital region, utilizing a variety of radiograph machines, and the primary input data was in that sense notably heterogeneous. This diversity may facilitate a broader application of our findings. However, we acknowledge that additional research in clinical settings is essential to enable the clinical use of the model. Furthermore, we were diligent in ensuring that our images did not feature overrepresented elements unrelated to the subject under examination, such as casts or hospital labels in the context of disease cases. We did not have a separate validation set to test DL model’s performance which is an obvious limitation in our study and may have resulted in an overfitted DL model. The use of separate validation set would not have been feasible regarding our binary outcome in Aiforia platform. However, in Aiforia platform, the algorithm learning process prematurely stops if the learning curve enters the plateau phase, and AI engines are unable to learn anything significantly new. Hence, early stopping is a build-in feature to prevent overfitting. In addition, parameter “iterations without progress” was kept as default (100 iterations), thus avoiding any extra iterative steps in the training process that may lead towards overfitting. Total area error was not zero in our training set which also argues against overfitted algorithm.

In conclusion, our DL model may be of clinical use in the future to assist in screening for this rare disease. There are two relevant problems regarding diagnostics of AVN of the lunate. Many general practitioners do not know that this disease exists and hence would not suspect it, and stage I is not visible in radiographs to the human eye. Our model can hopefully overcome both problems in the future. As usual, additional training and testing data are of great value when developing DL models for a rare condition. In particular, more data are needed to determine if the model can truly differentiate Lichtman stage I AVN from radiographs. External validation of the model and a prospective pilot study are needed to determine if the model works in different settings and can be generalized into clinical scenarios.

## Data Availability

Under the terms of our Institutional Review Board approval, the retrospective data used in this study, from Helsinki University Hospital, cannot be released to protect patient confidentiality.
